# Relationship between indirect genetic effects for growth, environmental enrichment, coping style and sex with the serum metabolome profile of pigs

**DOI:** 10.1038/s41598-021-02814-x

**Published:** 2021-12-03

**Authors:** Elda Dervishi, Inonge Reimert, Lisette E. van der Zande, Pramod Mathur, Egbert F. Knol, Graham S. Plastow

**Affiliations:** 1 Livestock Gentec, University of Alberta, 116 St and 85 Ave, Edmonton, AB T6G 2R3 Canada; 2 Adaptation Physiology Group, Department of Animal Sciences, Wageningen University and Research, P.O. Box 338, 6700 AH Wageningen, The Netherlands; 3 Topigs Norsvin Research Center B.V, 6640 AA Beuningen, The Netherlands

**Keywords:** Animal physiology, Metabolomics, Zoology

## Abstract

Including Indirect Genetic Effects (IGE) in breeding programs to reduce aggression in group housed animals has been proposed. However, the effect of selection for IGE for growth on animal metabolism and physiology is unknown. The purpose of this study was twofold: (1) To investigate the effects of this new breeding method along with two housing (barren and straw), coping style (high and low resisters) and sex (female and castrated males) options on the metabolome profile of pigs. (2) To identify and map biological processes associated with a regrouping test at 9 weeks of age. We used Nuclear Magnetic Resonance to quantify 49 serum metabolites at week 8, 9 and 22. Also, we quantified 3 catecholamines (tyramine, epinephrine, phenylethylamine) and serotonin and three water soluble vitamins (B2, B5 and B7). Overall, no significant differences were observed between negative and positive IGE animals. The magnitude of change (delta) of many metabolites as a response to the regrouping test was significantly affected by IGE, especially that of the amino acids (P < 0.05), being greater in positive IGE pigs. The regrouping test was associated with alteration in glycine, serine and threonine metabolism. In conclusion positive and negative IGE animals respond differently to the regrouping test.

## Introduction

Pork consumers are increasingly demanding good pork quality together with high animal welfare standards. Traits like behavior in relation to housing types, social interaction in group housed pigs, and different stress factors, are becoming economically important in breeding programs. Both environment and genetics have an impact on behavior and social interaction in group housed pigs. Modern pig production systems include regrouping of the animals at different stages e.g. weaning, and finishing stage. When regrouping occurs, pigs need to cope with abrupt changes in food supply, housing conditions and social environment. Animals adopt different coping strategies in response to these environmental stresses^1^. In relation to coping strategy, the backtest had been used to categorize pigs as high or low resisters. High-resisters (HR) pigs are characterized by greater number of struggles and vocalizations, when compared with low-resisters (LR)^1–3^. Moreover, pigs classified as HR and LR are characterized by differences in behavioral, physiological and endocrine responses to conflict situations^2,3^.

Current intensive indoor systems limit a pig’s ability to perform rooting and chewing behaviors leading to boredom, frustration and to redirect exploratory behavior towards pen mates^4^ resulting in competition, aggression, tail biting and other negative social interactions. The positive impact of environmental enrichment on pig behavior has been extensively studied and reviewed^5–7^. Genetics also plays an important role in the behavior of animals. Indirect Genetic Effect (IGE) describes the impact of an individual on trait values of other individuals and was first introduced by Griffing^8^. Including IGE in breeding programs to reduce aggression in group housed animals has been proposed^9–12^. Pigs selected for a positive effect on the growth of their group members (positive IGE pigs) performed less non-reciprocal biting behavior and less aggression in a regrouping test, and less biting on enrichment material^11,13^. In addition, positive IGE pigs were less fearful than negative IGE pigs as determined by several novelty tests^14,15^. The response of pigs in novelty tests seems to depend also on their sex, females being less fearful than castrated males^14^. Female pigs have been found to have lower leukocytes and basal cortisol but higher haptoglobin concentrations when comparing to castrated males^15^, suggesting physiological differences between females and castrated males.

Some evidence showed that at weaning the weight of positive and negative IGE pigs is not significantly different^5^. However, opposite to expectation, positive IGE animals grew slower in the first 4 days after weaning than negative IGE pigs^16^. Furthermore, the positive IGE animals had lower carcass weight and less muscle depth^5^. At present, it is not clear whether selection for IGE on growth, combined with different housing conditions, sex and coping strategy, has any negative or positive impact on the animals’ metabolism, physiology or performance.

Metabolomics, although relatively new in animal sciences, has also shown great potential to identify indicators of economically important traits such as feed efficiency^17^. Metabolomics approaches are expected to result in a better understanding of the physiological mechanisms underlying the traits of interests, as well as for pleiotropic effects and to predict which traits may be affected by the selection performed on another trait.

Based on the previous literature published using the same pigs and experimental design it has reported that pigs with positive and negative IGE differ in their behaviors, immunological status, and carcass traits suggesting differences in the metabolism. Therefore, in the present study we hypothesized that pigs selected for positive or negative IGE differ in their metabolome profile. The purpose of this study was two-fold: (1) To investigate the effects of IGE for growth, housing conditions, sex and coping style on the serum metabolome profile of pigs using a targeted metabolomics approach. (2) To identify and map biological processes associated with IGE for growth and a regrouping test.

## Results

The effect of time was significant for all metabolites except for ethanol and dimethyl sulfone concentration pointing to an effect of age on metabolite concentration. Batch was the second factor which affected a great number of metabolites and their ratios (forty-six) pointing to an effect of the season.

### The effect of indirect genetic effects on growth on metabolite concentration

Overall, no significant differences in the concentration of the metabolites were observed between negative and positive IGE animals. Negative IGE animals tended to have greater ratio of L-valine/isobutyric acid (69.30 ± 1.59 vs 65.20 ± 1.77; P = 0.09), L-leucine/isobutyric acid (39.70 ± 0.96 vs 37.0 ± 1.06; P = 0.07) and isoleucine/isobutyric acid (29.30 ± 0.70 vs 27.30 ± 0.78; P = 0.08). These ratios decreased over time reaching the lowest values at week 22 (Supplementary Fig. S1).

The magnitude of change (delta) of many metabolites as a response to regrouping was significantly affected by IGE class, especially that of the amino acids (P < 0.05). The significant metabolites are shown in Table 1. Negative IGE animals had a smaller delta of amino acids (AA) such as L-alanine, L-asparagine, L-aspartic acid, isoleucine, L-proline, L-serine and total glucogenic AA when compared to positive IGE animals (Table 1; P < 0.05). After regrouping, L-lysine, L-glycine and the sum of branched-chain amino acids (BAA), increased in negative IGE but decreased in positive IGE pigs (Table 1; P < 0.05). Moreover, negative IGE animals had a smaller delta of D-glucose, citric acid, choline, L-aspartic acid/L-glutamine ratio, creatine and creatine/creatinine ratio (Table 1; P < 0.05).

**Table 1:** Least squared means and SE of the magnitude of change (delta) of metabolite concentrations of pigs that have an estimated relative positive genetic effect or negative genetic effect (IGE) on the growth of their pen mates after regrouping at 9 weeks of age.

Metabolite µM	Negative IGE	Positive IGE	P value
L-alanine	− 231.61 ± 44.31	− 386.61 ± 49.60	0.006
L-asparagine	− 17.73 ± 5.21	− 34.19 ± 5.84	0.005
L-aspartic acid	− 8.54 ± 1.83	− 16.35 ± 2.10	0.003
L-isoleucine	− 23.40 ± 7.41	− 43.90 ± 8.13	0.009
L-proline	− 65.90 ± 17.31	− 125.62 ± 20.10	0.01
L-serine	− 13.69 ± 7.21	− 51.18 ± 8.17	0.0001
Glucogenic AA	− 370.0 ± 151.0	− 928.0 ± 165.0	0.0002
L-lysine	62.92 ± 20.60	− 1.65 ± 22.80	0.004
L-glycine	20.80 ± 56.69	− 188.90 ± 59.50	< 0.0001
BAA	37.20 ± 28.89	− 48.0 ± 27.81	0.003
D-glucose	− 309.17 ± 244.26	− 974.93 ± 264.40	0.004
Citric acid	− 58.1 ± 9.39	− 80.0 ± 10.18	0.01
Choline	− 3.92 ± 1.17	− 6.92 ± 1.28	0.02
L-aspartic acid/L-glutamine	− 1.57 ± 0.57	− 2.81 ± 0.64	0.04
Creatine	91.5 ± 19.9	163.1 ± 22.7	0.009
Creatine/creatinine	1.03 ± 0.24	2.03 ± 0.27	0.002

Multivariate analysis, partial least-squares discriminant analysis (PLS-DA) showed that positive and negative IGE animals do not display differences in their metabolite profiles, but a clear effect of time was observed, with samples collected at week 8, 9, and 22 clustering separately (Supplementary Fig. S2).

**Figure 1:**
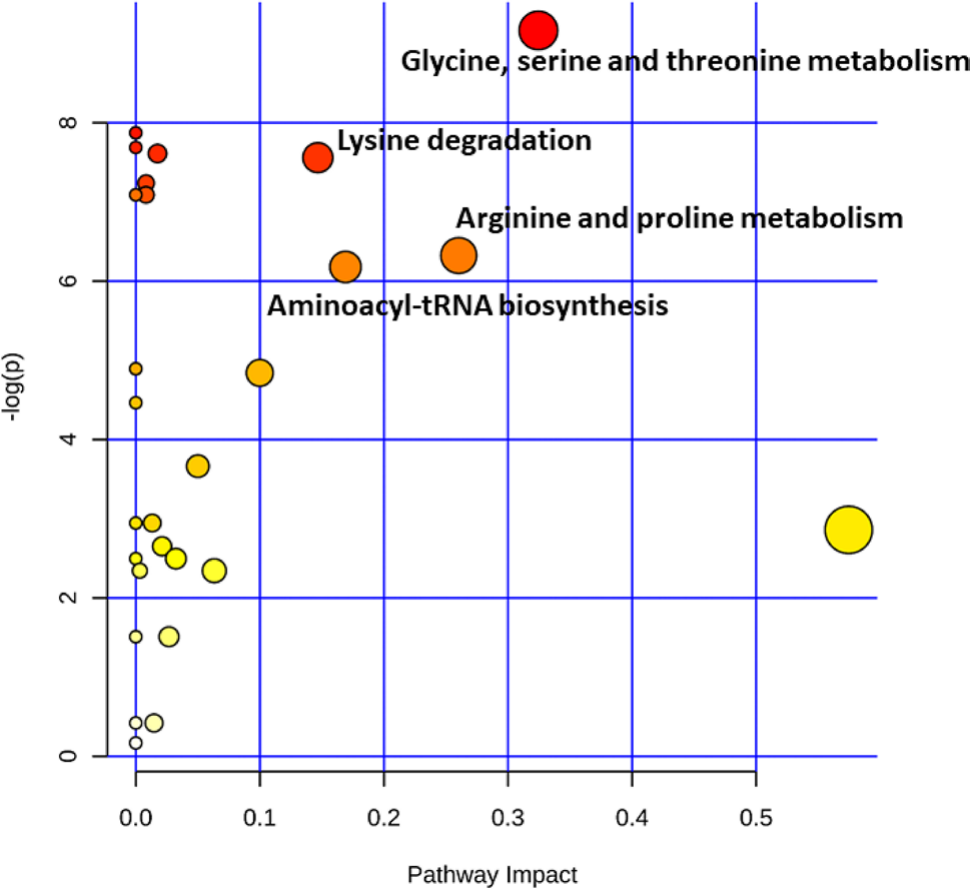
The results of pathway analysis depicting the metabolome view, displaying all the matched pathways as circles. The color is based on its p value and size of each circle on its pathway impact value.

Pathway analysis revealed that the top ranked pathways associated with the response to the regrouping test were: glycine, serine and threonine metabolism (compound members: L-serine, choline, L-glycine, L-aspartic acid and creatine), arginine and proline metabolism (compound members: L-arginine, L-aspartic acid, L-glutamine, L-proline, creatine), and L-lysine degradation (compound members: L-aspartic acid and L-lysine; Fig. 1).

Correlation analysis showed no significant correlation between the delta of metabolites and the delta of haptoglobin, IgG, IgM and leukocytes (P > 0.05).

### Housing

Housing significantly affected seven metabolites. Overall, pigs housed in the enriched environment had a greater concentration of creatinine, D-glucose, dimethyl sulfone, ethanol and L-tyrosine when compared with barren housed pigs (P < 0.05). On the other hand, enriched housed pigs had lower mannose and 3-methyl-2-oxovaleric acid concentration when compared with those in the barren environment (P < 0.05; Table 2).

**Table 2:** Least squared means and SE of the concentration of significant serum metabolites in animals housed in enriched and barren environments at week 8, 9 and 22.

Metabolites µM	Enriched	Barren	P value
Creatinine	94.20 ± 1.08	90.40 ± 1.18	0.01
D-glucose	5533 ± 65.80	5340 ± 72.20	0.04
Dimethyl sulfone	16.40 ± 0.90	13.70 ± 0.99	0.04
Ethanol	20.0 ± 0.69	17.70 ± 0.74	0.003
L-tyrosine	124 ± 1.90	118 ± 2.12	0.04
Mannose	52.60 ± 1.24	56.60 ± 1.42	0.02
3-Methyl-2-oxovaleric acid	7.47 ± 0.16	8.07 ± 0.17	0.008

The delta of the metabolites 2-hydroxybutyrate, isobutyric acid, L-glutamine and vitamin B5 was positive in animals housed in the barren environment, while it was negative in animals housed in the enriched environment after the regrouping test (Table 3; P < 0.05).

**Table 3:** Least squared means and SE of the significant magnitude of change (delta) of metabolite concentration of pigs that were housed in enriched and barren environments after regrouping at 9 weeks of age.

Metabolite µM	Enriched	Barren	P value
2-Hydroxybutyrate	− 3.69 ± 1.79	0.63 ± 1.76	0.01
Isobutyric acid	− 0.32 ± 0.43	1.18 ± 0.42	0.001
L-glutamine	− 0.40 ± 0.54	1.49 ± 0.53	0.001
Vitamin B5	− 0.14 ± 0.04	0.08 ± 0.04	0.0001

### Interaction between IGE on growth and housing conditions on the delta of metabolites

The interaction between IGE and housing conditions affected the delta of the amino acid L-threonine and body weight at week 10. In the barren environment negative IGE animals had a positive delta of L-threonine meanwhile positive IGE pigs showed a negative delta (Supplementary Fig. S3; P < 0.05). Overall, there was no significant difference in body weight between positive and negative IGE pigs, however at week 10, one week after regrouping test, positive IGE pigs housed in the barren environment had a lower body weight when compared with positive IGE pigs housed in the enriched environment (Supplementary Fig. S3; P < 0.05).

### Coping style

**Figure 2:**
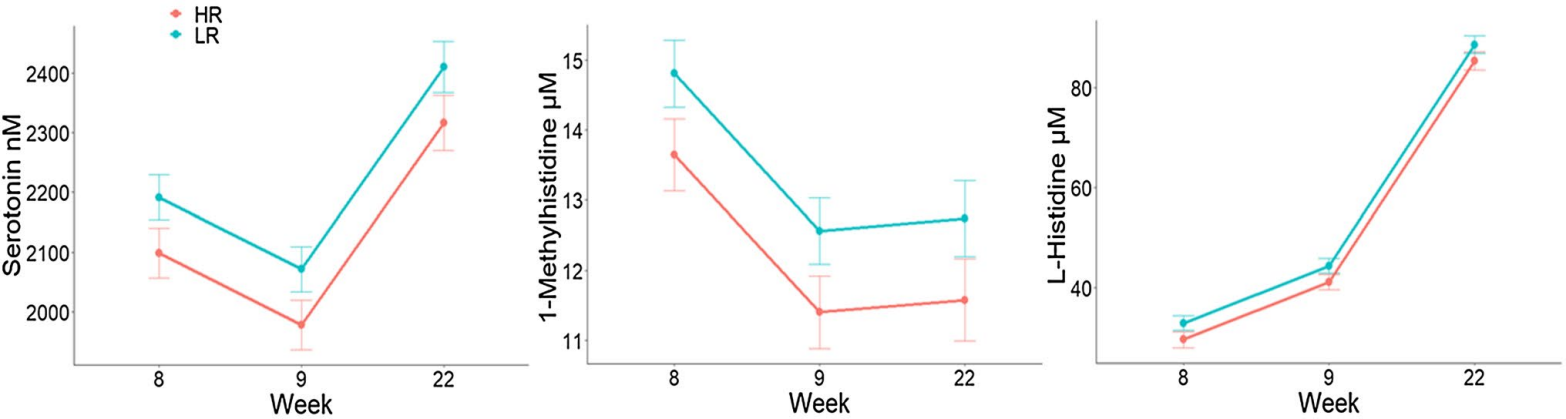
Least squared means and SE of serum metabolites and serotonin concentration in pigs with a high resisting (HR) and low resisting (LR) coping style at week 8, 9 and 22.

High resister pigs had an overall lower concentration of serotonin (P = 0.03), 1-methylhistidine (P = 0.02) and L-histidine (P = 0.04) when compared to LR (reactive) pigs (Fig. 2).

Acute stress due to regrouping had a larger impact on LR pigs. Pigs that were classified as HR had lower delta values of L-histidine, L-ornithine, ketoleucine, but greater delta values of vitamin B7, L-phenylalanine and L-tyrosine (Table 4; P < 0.05) when compared with pigs classified as LR.

**Table 4:** Least squared means and SE of the magnitude of change (delta) of metabolite concentrations in pigs with high resisting (HR) and low resisting (LR) coping styles after regrouping at 9 weeks of age.

Metabolite µM	HR	LR	P value
L-histidine	5.66 ± 2.32	15.81 ± 2.01	0.001
Ketoleucine	− 0.25 ± 0.44	0.93 ± 0.41	0.005
L-ornithine	− 3.34 ± 16.8	36.27 ± 15.1	0.04
L-phenylalanine	− 14.77 ± 3.39	4.93 ± 2.95	0.02
L-tyrosine	− 23.26 ± 5.32	− 9.35 ± 4.71	0.04
Vitamin B7	− 0.0035 ± 0.0008	0.0010 ± 0.0007	0.01

### Sex

**Figure 3:**
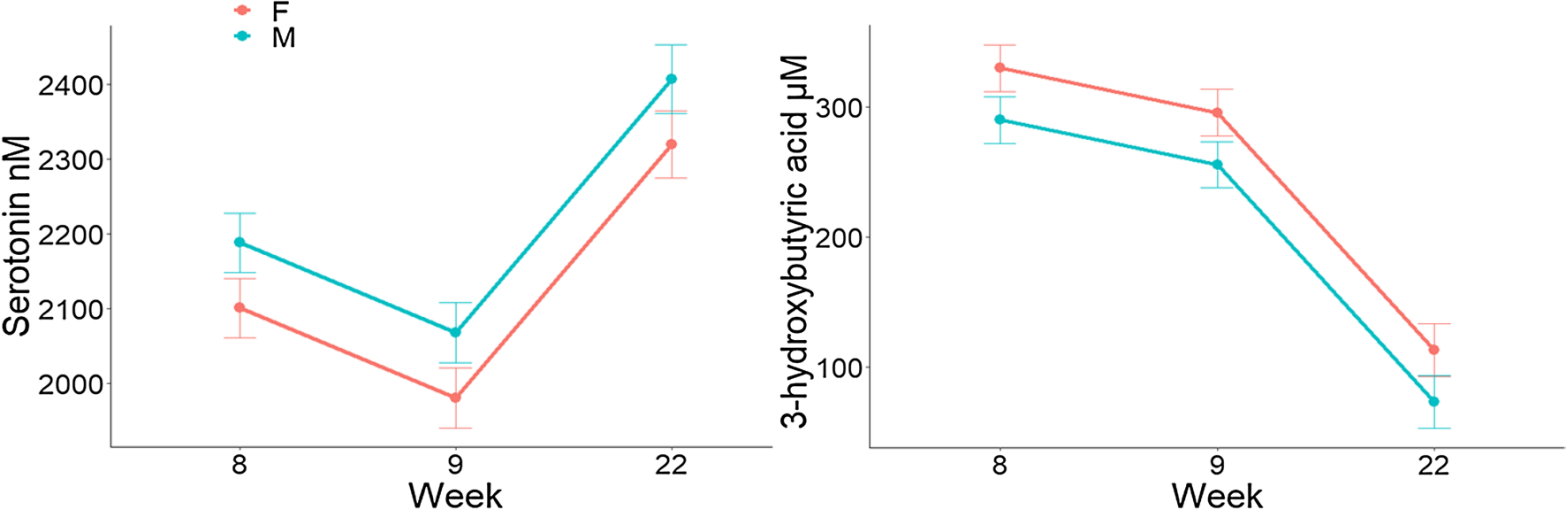
Least squared means and SE of serum 3-hydroxybutyric acid and serotonin concentrations in female (F) and castrated male (M) pigs at week 8, 9 and 22.

Overall, female pigs had a greater concentration of serum 3-hydroxybutyric acid (BHBA; P = 0.01), and a lower serum serotonin concentration (P = 0.05) when compared to castrated male pigs (Fig. 3). In addition, females tended to have greater L-lactic acid when compared to castrated males (7973 ± 181 vs 7502 ± 182 µM; P = 0.06). After regrouping, aspartic acid decreased to a smaller extent in female animals when compared to castrated males (− 9.42 ± 1.92 vs − 15.47 ± 1.93 µM; P < 0.05).

Concentration of serum serotonin was negatively correlated with L-lactic acid (R = − 0.24, P < 0.001).

## Discussion

In this study we investigated the effect of divergent selection for IGE on growth, environmental enrichment, coping style and sex on the metabolome profile of pigs using a targeted metabolomics approach. As was expected, time of sampling affected the concentration of all, except for two, metabolites, pointing to an effect of age on metabolite concentration. Age-related metabolite changes have been previously reported in human plasma^18^.

Overall, no significant differences in the concentration of the metabolites were observed between negative and positive IGE animals suggesting that this method of selection doesn’t impact the metabolic profile of pigs in this study. Positive IGE animals tended to have lower ratios of L-valine/isobutyric acid, L-leucine/isobutyric acid and isoleucine/isobutyric acid when compared with negative IGE animals. Branched-chain volatile fatty acids including isobutyric acid are built up from the degradation products of branched amino acids and it has been suggested to be indicative of better utilization of dietary protein by the microbiota^19^. However, our results showed only a tendency which deserve further future investigation.

Positive and negative IGE groups responded differently to acute stress induced by regrouping test. The extent to which the concentration of many metabolites changed after the regrouping test, especially that of the amino acids, D-glucose, citric acid, and creatine was greater in positive IGE pigs. These results suggest two possibilities: Either the positive IGE pigs were mounting an immune response or they were temporarily in an energy deficiency state after the regrouping test. It was proposed that negative IGE pigs might have a more active immune system which, could lead to an increased need of amino acids e.g. for synthesis of haptoglobin^20^. To test this hypothesis, we conducted correlation analysis between the delta of metabolites and delta of haptoglobin, IgG, IgM and leukocytes, reported by Reimert et al.^20^. No significant correlation was found; therefore, it appears that the magnitude of change after the regrouping test might not be related with mounting an immune response. Another possible explanation of our results might be that the animals were temporarily in an energy deficiency status after a stressful event such as the regrouping test.

The delta of amino acids and other metabolites was altered in both positive and negative IGE animals. However, the extent of increase or decrease was more pronounced in positive IGE animals. For example, the extent to which glucogenic amino acids, D-glucose and citric acid decreased in serum and the extent to which creatine increased was greater in positive IGE animals. During energy deficiency, glycolysis, lipolysis and proteolysis processes develop simultaneously. During proteolysis, muscle tissue is used as a source of amino acid. Glucogenic amino acids are converted into glucose which is used as energy source. In our study, glucogenic amino acids and D-glucose decreased to a greater extent in positive IGE pigs suggesting that positive IGE pigs were in a more pronounced state of energy deficiency when compared to negative IGE pigs.

Furthermore, pathway analysis showed that the top ranked pathway associated with the response to regrouping was glycine, serine and threonine metabolism. This pathway has been reported to be genetically correlated with the average daily gain in pigs^21^. The amino acid L-glycine decreased in positive IGE animals after regrouping. L-Glycine acts as a precursor for creatine, therefore the decrease in L-glycine in positive IGE pigs could explain the increase in creatine in these animals after the regrouping test. Creatine is synthesized in the liver, kidney and pancreas^22^. Approximately 95% of creatine is stored in skeletal muscle in the form of phosphocreatine. During energy demand, phosphocreatine transfers its phosphate group to ADP to form ATP and creatine. Creatine is then spontaneously transformed to creatinine, transported out of skeletal muscle via the blood and eliminated by the kidneys^23^. The creatine/creatine-phosphate system plays an important role in the storage and transmission of phosphate-bound energy. The greater increase in creatine concentration and the ratio creatine/creatinine, might reflect a greater energy demand of positive IGE animals to cope with the regrouping test.

In commercial settings, pigs are often relocated and mixed with other unknown pigs at weaning and/or at grower and finisher stages. Behavioral studies conducted by Reimert et al.^15^ demonstrated that pigs with a positive IGE were faster to interact with ropes provided in a novel rope test than pigs with a negative IGE. Moreover, in a more recent study on these same animals, Camerlink et al.^16^ reported that positive IGE pigs had a greater latency to start eating and a lower weight gain over the first 4 days after weaning. Therefore, it is plausible that at regrouping, animals with positive IGE will explore the new environment first and spend more time establishing social relationships rather than approaching feed and maybe eating less, which in turn could have led to a temporary energy deficiency status as a response to regrouping.

A wealth of research has demonstrated that environmental enrichment exerts biological effects in many species, most of which are beneficial, and keeping the animals in a barren environment invokes chronic stress^1,24^. Environmental enrichment has been shown to increase the concentration of brain-derived neurotrophic factor in pigs provided with foraging enrichment^25^ and decrease levels of lactate^7^. In our study enriched pigs had greater concentrations of serum L-tyrosine when compared with animals in the barren environment. L-tyrosine is the precursor of the catecholamines and neurotransmitters^26^ and supplementation with tyrosine may reduce stress responses in swine^27^. In a previous study that was carried out using the same pigs as the current one, enriched housed pigs were less stressed^20^ when compared with those animals housed in a barren environment. Therefore, a link between straw provision, stress response and tyrosine can not be excluded and deserves further investigation. This result also suggests that supplementing L-tyrosine might be a feeding strategy to reduce the stress response in pigs that are raised in barren environment.

Enriched housed pigs had lower 3-methyl-2-oxovaleric acid when compared with barren housed pigs. This compound is a keto-acid that induces acidosis, that arises from the incomplete breakdown of branched-chain amino acids^28^. To our knowledge, reports about 3-methyl-2-oxovaleric acid in pigs are absent. Enriched housed pigs could had a better utilization of branched amino acids and energy status, as also shown by the greater D-glucose concentration. Also, during the regrouping test (providing acute stress) housing pigs in an enriched environment could results in a better utilization of amino acids. In support of this idea, we found that delta of 2-hydroxybutyrate decreased in animals housed in the enriched environment. 2-hydroxybutyrate is produced by amino acid catabolism (L-threonine and L-methionine). In humans, 2-hydroxybutyrate appears at high concentrations in situations related to deficient energy metabolism and has been reported as an early marker for impaired glucose regulation^29^. We think that providing straw has a positive effect on the ability of pigs to cope with acute stress during regrouping with other unknown pigs.

The molecular basis for how straw induces modulation of metabolites or other compounds are unknown, however there is some evidence that providing enrichment modulates the concentration of some hormones and metabolites^7,25^. The purpose of providing enrichment (straw) is to serve as stimulus for pigs to perform exploratory, manipulative and rooting behavior. As pigs perform their natural behaviors, they also get more active which in turn leads to mobilization of different energy sources (carbohydrates, lipids and proteins). This could be one of the mechanisms that “kick start” the changes we found in the concentration of metabolites which are the intermediate or end product of the metabolism.

When in stressful situations animals can adopt coping strategies. Immunological differences between HR and LR pigs have been previously described^20,30^. For example, HR pigs were reported to have greater complement activity via the alternative pathway (APW), therefore a more active innate immune system than LR pigs^20^. In our study HR, had lower serum concentration of serotonin, histidine and 1-methylhistidine when compared to LR pigs. Interestingly, Hessing et al.^31^ reported that a difference in balance between T-helper 1 and T-helper 2 cells was underlying the difference between HR and LR pigs. Peripheral serotonin exerts pro-inflammatory as well as anti-inflammatory responses^32,33^. Immune cells have the capacity to synthesize, transport, store, degrade and respond to serotonin which exerts an important role in the balance of T cell subsets^32,33^. The differences in serum serotonin concentration between HR and LR suggest a difference in the serotonergic system between HR and LR pigs which is in agreement with Koolhaas et al.^34^.

In our study, female pigs were found to have lower serum serotonin. Differences in the rates of brain serotonin synthesis between males and females have been reported in humans^35^ and rodents^36^. In addition, female pigs had greater concentration of BHBA and tended to have greater concentration of L-lactic acid during the experiment. BHBA is a ketone body and together with the accumulation of lactic acid can lead to reduced serum pH. Interestingly, pigs and chickens fed acidified diets had depressed levels of serotonin in the brain^37,38^. In addition, it was proposed that lactic acid increases serotonin uptake in human blood platelets in vitro by 40% thereby decreasing serotonin concentration in serum^39^. In our study serum serotonin was negatively correlated with L-lactic acid, therefore, a connection between serotonin, lactic acid and BHBA cannot be ruled out. We speculate that BHBA and lactic acid concentration could be one of the underlying physiological differences in serotonin concentration between females and castrated males.

In conclusion selection for positive or negative IGE for growth does not impact the metabolic profile and weight of the animals used in this study. However, animals selected for IGE responded differently to regrouping. The regrouping test (providing acute stress) had a greater impact on the metabolic profile of positive IGE animals. However, no differences in body weight were observed. The practical implication of this study is that while selection for IGE doesn’t have a negative impact on serum metabolites concentration, regrouping (mixing) is a critical process which can seriously impact the performance of the animals. More research is necessary to determine whether selection for more than one generation on IGE for growth, has any impact on metabolism, physiology, performance and response to acute stress. To our best knowledge this is one of the first studies to report that housing enrichment modulates metabolites concentration in pigs. Besides, differences in serum serotonin concentration, suggest a difference in the serotonergic system between HR and LR pigs and between females and castrated males.

## Methods

All experimental procedures were approved by the University of Alberta Animal Policy and Welfare Committee for Livestock as category “A” level of invasiveness (no animal manipulation as the animal work had already been completed and the present study involved only laboratory analysis). Animals had been cared for in accordance with the recommendations in the European Guidelines for accommodation and care of animals. The protocol was approved by the Institutional Animal Care and Use Committee of Wageningen University (Protocol Number: 2010055f.). All procedures were carried in accordance with the Animal Research: Reporting of In Vivo Experiments (ARRIVE) guidelines^40^.

This study was part of the project ‘Seeking Sociable Swine’ designed to study the opportunities to improve social interactions among pigs by incorporating IGE in the breeding program and investigate the implications of this selection method for pig behavior and welfare. Details of the materials and methods are described by Camerlink et al*.*^5^, Camerlink et al.^13^, Reimert et al*.*^14^ and Reimert et al.^15^. The current study builds on the same experiment but it is focused on metabolite concentrations measured in serum and their association with IGE for growth, housing conditions, coping style, sex and response to the regrouping test. For the purpose of clarity some relevant details are provided below, but briefly.

### Animals

Pigs were born at the experimental farm of Topigs Research Center IPG in Beilen, the Netherlands. Until weaning, they were housed in standard lactation pens of 3.8 m^2^. At 3 days of age males were surgically castrated but tails were not docked. At four weeks of age, the piglets were weaned, and a total of 480 offspring Tempo × Topigs-20 pigs were introduced in five different batches (96 pigs/batch). Pigs were housed until slaughter at 23 weeks of age in 16 pens of 6–7 m^2^ located in one room per batch at the animal facility of Wageningen University, Wageningen, the Netherlands. Each pen consisted of six unrelated pigs, three females and three males. In addition, in each pen there were at least two pigs with a high-resisting (HR) coping style and two with a low-resisting (LR) coping style^4,20^. Half of the pigs were housed in a barren environment, and the other half in a straw enriched environment (Supplementary Material and Method). All pigs were subjected to a regrouping test at week 9 of life to induce acute stress and to simulate a similar procedure which takes place in a farm setting. The details of behavioral tests (coping style classification and regrouping test) are presented in Supplementary Material and Method.

A total of 180 animals were included in the metabolomics analysis (animals from batch 1 and batch 2), balanced regarding sex and coping style, selected for positive IGE (n = 78) and negative IGE (n = 102) for growth of their pen mates and housed in barren or straw-enriched pens. The number of the animals in each group are summarized in Supplementary Table S1.

### Blood collection and weight

Blood was collected three times; once in the week before regrouping (8 weeks of age), three days after regrouping (9 weeks of age), and at 22 weeks of age. Blood was taken by venipuncture from the jugular vein and collected in serum separating tubes and incubated for one hour at 37 °C after which they were centrifuged at 3200 rpm for 12 min at 20 °C. Sera were stored at − 80 °C until further analysis.

For this study body weight records at 10, 17 and 23 weeks of age were considered in the statistical analysis.

### Metabolomics analysis

Metabolomics analysis was performed at The Metabolomics Innovation Centre (TMIC) at University of Alberta following established protocols. Serum samples were used for Nuclear Magnetic Resonance (NMR) analysis to measure the concentration of 49 metabolites including amino acids, sugars, alcohols, organic acids, amines, TCA cycle intermediates, and short chain fatty acids.

Because catecholamines are an essential component of the stress responses of animals and serotonin is a neurotransmitter involved in regulation of emotional states such as fear and aggression we quantified 3 catecholamines (tyramine, epinephrine, phenylethylamine) and serotonin. In addition, three water soluble vitamins (B2, B5 and B7) which are involved in regulation of carbohydrate, fat and protein metabolism^41^ were quantified.

Three amino acid indexes were calculated: the sum of glucogenic, ketogenic, and branched AA. Glucogenic AA were calculated as the sum of all the amino acids, except L-leucine and L-lysine. Ketogenic AA were calculated as the sum of L-leucine and L-lysine. Branched AA were calculated as the sum of L-valine, L-leucine, and L-isoleucine. In addition, the sum of ketone bodies and the ratios L-valine/isobutyric acid, L-leucine/isobutyric acid, L-isoleucine/isobutyric acid, L-aspartic acid/glutamic acid, creatine/creatinine were calculated because they are involved in the same metabolic pathway. The number of the variables that were included in the analysis are summarized in Supplementary Table S2.

#### Nuclear magnetic resonance compound identification and quantification

All serum samples were thawed on ice before use. A deproteinization step, involving ultra-filtration was introduced in the protocol to remove serum proteins. Prior to filtration, 3 KDa cut-off centrifugal filter units (Amicon Microcon YM-3) were rinsed five times each with 0.5 mL of H_2_O and centrifuged (10,000 rpm for 10 min) to remove residual glycerol bound to the filter membranes. Aliquots of each serum sample were then transferred into the centrifuge filter devices and spun (10,000 rpm for 20 min) to remove macromolecules (primarily protein and lipoproteins) from the sample. The filtrates were collected and the volumes were recorded and samples diluted to 200 µL with 50 mM potassium salt buffer (pH 7) as needed. The dilution factor was recorded and metabolite concentrations were corrected accordingly in the subsequent analysis. Subsequently, 50 µL of a standard buffer solution (54% D_2_O: 46% 250 mM K_2_HPO_4_ + KH_2_PO_4_ pH 7.0 v/v containing 5 mM DSS (2,2-dimethyl-2-silcepentane-5-sulphonate), 5.84 mM 2-chloropyrimidine-5 carboxylate, and 0.1% NaN_3_ in H_2_O)) was added to the sample. The sample (250 µL) was then transferred to a 3 mm SampleJet NMR tube for subsequent spectral analysis. All 1H-NMR spectra were collected on a 700 MHz Avance III (Bruker) spectrometer equipped with a 5 mm HCN Z-gradient pulsed-field gradient cryoprobe. NMR pulse sequence parameters were as following: acquisition time 4 s, mixing time 100 ms, recycle delay 10 ms, saturation delay 990 ms, transients (700 MHz) 128, steady state scans 8 and presaturation field width 80 Hz. 1H-NMR spectra were acquired at 25 °C using the first transient of the NOESY pre-saturation pulse sequence (noesypr1d), chosen for its high degree of quantitative accuracy^42^. All Free Induction Decays were zero-filled to 250 K data points. The singlet produced by the DSS methyl groups was used as an internal standard for chemical shift referencing (set to 0 ppm) and for quantification all 1H-NMR spectra were processed and analyzed using the online Bayesil software package^43^. The spectral fitting for each metabolite was done using the standard serum metabolite library. Most of the visible peaks were annotated with a compound name.

#### Catecholamines and serotonin quantification

Catecholamine quantification was carried out following the protocol reported by Zheng et al.^44^ All serum samples were thawed on ice in the dark before use and transferred to a 96-well filter plate. Ten μL of the internal standard mixture (ISTD) solution and 25 μL of samples (PBS for “zero” samples, calibration curve standards, and serum samples) were pipetted directly onto the center of each well. The whole plate was evaporated under nitrogen flow for 45 min to dry. Fifty μL of phenylisothiocyanate (PITC) derivatization solution was then added to each well, and the reaction was kept at room temperature for 20 min. The samples were again dried in a nitrogen evaporator for 90 min to remove the liquid, especially the excess PITC, followed by the addition of 75 μL of methanol containing 5 mM ammonium acetate. The plate was covered and shaken at 450 rpm for 30 min at room temperature, and then spun by centrifuge for 15 min at 1200 rpm. To each well of the collection plate, 75 μL of water was added and mixed thoroughly, and 50 μL was injected for LC–MS/MS analysis. An Agilent 1100 series HPLC system (Agilent, Palo Alto, CA) and an Agilent reversed-phase Zorbax Eclipse XDB C18 column (3.0 mm × 100 mm, 3.5 μm particle size, 80 Å pore size) with a Phenomenex (Torrance, CA, U.S.A.) SecurityGuard C18 pre-column (4.0 mm × 3.0 mm) were used for online LC–MS/MS with an AB SCIEX QTRAP® 4000 mass spectrometer (Sciex Canada, Concord, ON.). The controlling software for the LC–MS system was Analyst® 1.6.2. For the HPLC work, solvent A was 0.2% (v/v) formic acid in water, and solvent B was 0.2% (v/v) formic acid in ACN. The gradient profile for the HPLC solvent run was as follows: t = 0 min, 0% B; t = 0.5 min, 0% B; t = 5.5 min, 95% B; t = 6.5 min, 95% B; t = 7.0 min, 0% B; and t = 9.5 min, 0% B. The column oven was set to 50 °C. The flow rate was 500 μL/min, and the sample injection volume was 40 μL. The mass spectrometer was set to a positive electrospray ionization mode with multiple reaction monitoring (MRM). The IonSpray voltage was set at 5500 V and the temperature at 500 °C. The curtain gas (CUR), ion source gas 1 (GAS1), ion source gas 2 (GAS2) and collision gas (CAD) were set at 20, 40, 50 and medium, respectively. The entrance potential (EP) was set at 10 V. Likewise, the declustering potential (DP), collision energy (CE), collision cell exit potential (CXP), MRM Q1 and Q3 were optimized and set individually for each analyte and each corresponding deuterated ISTD^44^.

#### Vitamin quantification

Vitamins B2, B5 and B7 were quantified. Samples were thawed on ice, transferred to a 96-well plate and centrifuged at 10,000 rpm for 3 min. Ten µL of the ISTD solution was added to all wells of the plate except the blank well. A seven-point calibration curves was used for quantification. Three QCs (synthetic mixtures, low, medium and high) were used. Fifty µL of calibration standards was pipetted onto the center of wells starting from the lowest concentration to the highest concentration. Fifty µL QCs was added onto the center of wells starting from the lowest concentration to the highest concentration and 50 µL of sample was added onto the center of the remaining wells. In addition, 100 µL of extraction solvent (19 mg ammonium acetate in 50 mL HPLC grade methanol) was added to each well. The plate was covered with the plastic lid and shaken for 30 min at 150 rpm and centrifuged for 5 min at 500 × g. All the wells in the capture plate were diluted with 50 µL of HPLC grade water and sealed. The plate was shaken for 2 min at 150 rpm and run using LC–MS. The running time was 7 min. An Agilent 1100 series HPLC system (Agilent, Palo Alto, CA) and a Phenomenex (Torrance, CA, USA) Kinetex C18 (2.6 µm, 3.0 × 100 mm, 100A, S/N: 669679-8) with a Phenomenex SecurityGuard C18 pre-column (4.0 mm × 3.0 mm) were used for online LC–MS/MS with an AB SCIEX QTRAP® 4000 mass spectrometer (Sciex Canada, Concord, ON.). The controlling software for the LC–MS system was Analyst® 1.6.2. For the HPLC work, solvent A was 10 mM AmFA in water, and solvent B was 10 mM AmFA in 90:10 ACN:water. The gradient profile for the HPLC solvent run was as follows: t = 0 min, 0% B; t = 2.0 min, 30% B; t = 3.5 min, 60% B; t = 5.0 min, 75% B; t = 5.5 min, 75% B; t = 5.6 min, 0% B; and t = 7.0 min, 0% B. The column oven was set to 40 °C. The flow rate was 500 μL/min, and the sample injection volume was 5 μL. The mass spectrometer was set to a positive electrospray ionization mode with MRM. The IonSpray voltage was set at 5500 V and the temperature at 450 °C. The curtain gas, ion source GAS1, ion source GAS2 and CAD were set at 20, 40, 60 and medium, respectively. The EP was set at 10 V. Likewise, DP, CE, CXP, MRM Q1 and Q3 were optimized and set individually for each analyte and each corresponding deuterated ISTD.

### Statistical analysis

Univariate statistical analysis was performed using R (version 3.5.2)^45^. The response variables were the metabolite concentrations and weight. Statistical analysis for weight was carried out considering the animal weight at 10, 17 and 23 weeks of age. Prior to the analysis, when appropriate, metabolites were log transformed to obtain normality of residuals. Fixed effects were tested for their significance by fitting a Mixed Model for Repeated Measurements assuming a first-order autocorrelation variance–covariance structure (CorCAR1) which allows for unequally spaced time observations with the final model being:

$${\text{y}} = \mu + {\text{IGE}} + {\text{time}} + {\text{sex}} + {\text{batch}} + {\text{housing}} + {\text{coping style}} + {\text{IGE}}*{\text{housing}} + {\text{pen}} + {\text{e}},$$

where y is the response variable (metabolites and weight), IGE (positive or negative), time (week 8, week 9, week 22), sex (females and castrated males), batch (1 and 2), housing (barren and enriched), coping style (HR, LR), and housing condition and its interaction with IGE were included as fixed factors, pen was a random effect nested within IGE, and e refers to the common error term. To test differences between IGE classes, housing condition, coping style and sex the Least Square Means (LSMs) for each pair-wise comparison were estimated and adjusted using false discovery ratio (FDR). In addition, statistical analysis for weight were done separately for week 10, 17 and 23 with the Linear Mixed Model being:

$${\text{y}} = \mu + {\text{IGE}} + {\text{sex}} + {\text{batch}} + {\text{housing}} + {\text{coping style}} + {\text{IGE}}*{\text{housing}} + {\text{pen}} + {\text{e}},$$

where IGE class, sex, batch, housing condition, coping style and housing condition and its interaction with IGE, were included as fixed effects, pen as a random effect and e refers to the common error term.

To investigate physiological differences in response to the regrouping test, the delta of metabolites between week 9 and week 8 was calculated and analyzed with a Linear Mixed Model:

$${\text{y}} = \mu + {\text{IGE}} + {\text{sex}} + {\text{batch}} + {\text{housing}} + {\text{coping style}} + {\text{IGE}}*{\text{housing}} + {\text{pen}} + {\text{e}},$$

where IGE class, sex, batch, housing condition, coping style and housing condition and its interaction with IGE, were included as fixed effects, pen as a random effect and e refers to the common error term.

In addition, we performed correlations analysis between: 1-serum serotonin and lactic acid and 1- between the delta of metabolite concentrations and the delta of haptoglobin, IgG, IgM and leukocytes, reported by Reimert et al^20^. Significant results were considered if P ≤ 0.05 and P > 0.05 and ≤ 0.10 it was considered as a tendency.

Multivariate analysis was performed considering the metabolites’ concentration at week 8, 9 and 22 separately. Before multivariate analysis each metabolite concentration was adjusted for the significant fixed effect (sex, batch, housing and coping style) other than time and IGE. For each metabolite, the adjusted values were extracted and used for Partial Least Squares- Discriminant Analysis (PLS-DA). Multivariate analysis was performed using a web-based tool *MetaboAnalyst* (https://www.metaboanalyst.ca/) according to previously published protocols^46^.

To understand biological processes involved in the response to regrouping in the two IGE classes, pathway analysis was performed using the list of significant “delta” of metabolites and pathway-associated metabolite sets were selected as metabolite library. Metabolic pathway analysis calculates the impact on the pathways based on network topology analysis. For this analysis we used Homo sapiens (SMPDB) as the library because no library for pigs exists. In order to estimate the importance of a compound within a given metabolic network, we selected global test algorithm and relative-betweenness centrality which measures the number of shortest paths going through the node of interest^46^.

## Supplementary Information

Supplementary Figure S1.

Supplementary Figure S2.

Supplementary Figure S3.

Supplementary Material and Method.

Supplementary Table S1.

Supplementary Table S2.

## References

[1] Bolhuis JE, Schouten WGP, Schrama JW, Wiegant VM (2005). Behavioural development of pigs with different coping characteristics in barren and substrate enriched housing conditions. Appl. Anim. Behav. Sci..

[2] Hessing MJC, Hagelsø AM, Schouten WGP, Wiepkema PR, Van Beek JAM (1994). Individual behavioral and physiological strategies in pigs. Physiol. Behav..

[3] Koolhaas J (2008). Coping style and immunity in animals: making sense of individual variation. Brain Behav. Immunol..

[4] Prunier A, Heinonen M, Quesne H (2010). High physiological demands in intensively raised pigs: impact on health and welfare. Animal.

[5] Camerlink I, Bolhuis JE, Duijvesteijn N, van Arendonk JAM, Bijma P (2014). Growth performance and carcass traits in pigs selected for indirect genetic effects on growth rate in two environments. J. Anim. Sci..

[6] Mkwanazi MV, Ncobela CN, Kanengoni AT, Chimonyo M (2018). Effects of environmental enrichment on behaviour, physiology and performance of pigs: a review. Asian-Aust. J. Anim. Sci..

[7] Fàbrega E (2019). The effects of environmental enrichment on the physiology, behaviour, productivity and meat quality of pigs raised in a hot climate. Animals.

[8] Griffing B (1967). Selection in reference to biological groups I: individual and group selection applied to populations of unordered groups. Aust. J. Biol. Sci..

[9] Muir WM (1996). Group selection for adaptation to multiple-hen cages: selection program and direct responses. Poult. Sci..

[10] Bijma P, Muir WM, Ellen ED, Wolf JB, Van Arendonk JAM (2007). Multilevel selection 2: estimating the genetic parameters determining inheritance and response to selection. Genetics.

[11] Camerlink I, Turner SP, Bijma P, Bolhuis EJ (2013). Indirect genetic effects and housing conditions in relation to aggressive behaviour in pigs. PLoS ONE.

[12] Ellen ED (2014). The prospects of selection for social genetic effects to improve welfare and productivity in livestock. Front. Genet..

[13] Camerlink I, Ursinus WW, Bijma P, Kemp B, Bolhuis EJ (2015). Indirect genetic effects for growth rate in domestic pigs alter aggressive and manipulative biting behaviour. Behav. Genet..

[14] Reimert I (2013). Backtest and novelty behavior of female and castrated male piglets, with diverging social breeding values for growth. J. Anim. Sci..

[15] Reimert I, Rodenburg TB, Ursinus WW, Kemp B, Bolhuis JE (2014). Responses to novel situations of female and castrated male pigs with divergent social breeding values and different backtest classifications in barren and straw-enriched housing. Appl. Anim. Behav. Sci..

[16] Camerlink I, Ursinus WW, Bartels AC, Bijma P, Bolhuis JE (2018). Indirect genetic effects for growth in pigs affect behaviour and weight around weaning. Behav. Genet..

[17] Karisa B, Moore S, Plastow G (2014). Analysis of biological networks and biological pathways associated with residual feed intake in beef cattle. J. Anim. Sci..

[18] Vignoli A, Tenori L, Luchinat C, Saccenti E (2018). Age and sex effects on plasma metabolite association networks in healthy subjects. J. Proteome Res..

[19] Walsh AM, Sweeney T, Bahar B, Flynn B, O'Doherty JV (2013). The effects of supplementing varying molecular weights of chitooligosaccharide on performance, selected microbial populations and nutrient digestibility in the weaned pig. Animal.

[20] Reimert I, Rodenburg TB, Ursinus WW, Kemp B, Bolhuis JE (2014). Selection based on indirect genetic effects for growth, environmental enrichment and coping style affect the immune status of pigs. PLoS ONE.

[21] Dervishi E (2021). Heritability and genetic correlations of plasma metabolites of pigs with production, resilience and carcass traits under natural polymicrobial disease challenge. Sci. Rep..

[22] Brosnan JT (2009). Creatine synthesis is a major metabolic process in neonatal piglets and has important implications for amino acid metabolism and methyl balance. J. Nutr..

[23] Li J (2018). Creatine monohydrate and guanidinoacetic acid supplementation affects the growth performance, meat quality, and creatine metabolism of finishing pigs. J. Agric. Food Chem..

[24] Zonderland JJ (2008). Prevention and treatment of tail biting in weaned piglets. Appl. Anim. Behav. Sci..

[25] Rault JL, Lawrence AJ, Ralph CR (2018). Brain-derived neurotrophic factor in serum as an animal welfare indicator of environmental enrichment in pigs. Dom. Anim. Endocrin..

[26] Wu G (2009). Amino acids: metabolism, functions, and nutrition. Amino Acids.

[27] Adeola O, Ball RO (1992). Hypothalamic neurotransmitter concentrations and meat quality in stressed pigs offered excess dietary tryptophan and tyrosine. J. Anim. Sci..

[28] Przyrembel H (1979). Propionyl-CoA carboxylase deficiency with overflow of metabolites of isoleucine catabolism at all levels. Euro. J. Ped..

[29] Gall WE (2010). RISC Study Group: alpha-hydroxybutyrate is an early biomarker of insulin resistance and glucose intolerance in a nondiabetic population. PLoS ONE.

[30] Bolhuis JE, Parmentier HK, Schouten WGP, Schrama JW, Wiegant VM (2003). Effects of housing and individual coping characteristics on immune responses of pigs. Phys. Behav..

[31] Hessing MJC, Coenen GJ, Vaiman M, Renard C (1995). Individual differences in cell-mediated and humoral immunity in pigs. Vet. Immunol. Immunopath..

[32] Herr N, Bode C, Duerschmied D (2017). The effects of serotonin in immune cells. Front. Cardiovasc. Med..

[33] Wu H, Denna TH, Storkersen JN, Gerriets VA (2019). Beyond a neurotransmitter: the role of serotonin in inflammation and immunity. Pharmacol. Res..

[34] Koolhaas JM, de Boer SF, Coppens CM, Buwalda B (2010). Neuroendocrinology of coping styles: towards understanding the biology of individual variation. Front. Neuroendocrin..

[35] Nishizawa S (1997). Differences between males and females in rates of serotonin synthesis in human brain. PNAS.

[36] Dominguez R, Cruz-Morales SE, Carvalho MC, Xavier M, Brandao ML (2003). Sex differences in serotonergic activity in dorsal and median raphe nucleus. Physiol. Behav..

[37] Smulikowska S (2004). Effects of acidifier added to diets containing graded levels of tryptophan on growth performance, protein digestibility, and on brain serotonin level in broiler chickens. J. Anim. Feed Sci..

[38] Pastuszewska B, Tomaszewska-Zaremba D, Buraczewska L, Święch E, Taciak M (2007). Effects of supplementing pig diets with tryptophan and acidifier on protein digestion and deposition, and on brain serotonin concentration in young pigs. Anim. Feed Sci. Tech..

[39] Lingjaerde O (1985). Lactate-induced panic attacks: possible involvement of serotonin reuptake stimulation. Acta Psyc. Scand..

[40] Animal Research: Reporting of In Vivo Experiments (ARRIVE). https://arriveguidelines.org.

[41] Kennedy DOB (2016). Vitamins and the brain: mechanisms, dose and efficacy—a review. Nutrients.

[42] Saude EJ, Slupsky CM, Sykes BD (2006). Optimization of NMR analysis of biological fluids for quantitative accuracy. Metabolomics.

[43] Ravanbakhsh S (2015). Accurate, fully-automated NMR spectral profiling for metabolomics. PLoS ONE.

[44] Zheng J, Mandal R, Wishart DS (2018). A sensitive, high-throughput LC-MS/MS method for measuring catecholamines in low volume serum. Anal. Chim. Acta..

[45] R Development Core Team. R: A language and environment for statistical computing. Vienna, Austria: R Foundation for Statistical Computing. http://www.R-project.org (2008).

[46] Xia J, Wishart DS (2016). Using MetaboAnalyst 3.0 for comprehensive metabolomics data analysis. Curr. Prot. Bioinform..

