# Supplementary material for: Relationship between indirect genetic effects for growth, environmental enrichment, coping style and sex with the serum metabolome profile of pigs
Source: Sci Rep. 2021 Dec 3;11:23377. doi: 10.1038/s41598-021-02814-x (PMC8642533; doi:10.1038/s41598-021-02814-x)

**Supplementary Fig. S1**. Least squared means and SE of metabolites in animals that have an estimated relative positive genetic effect or negative genetic effect (IGE) on the growth of their pen mates at week 8, 9 and 22.


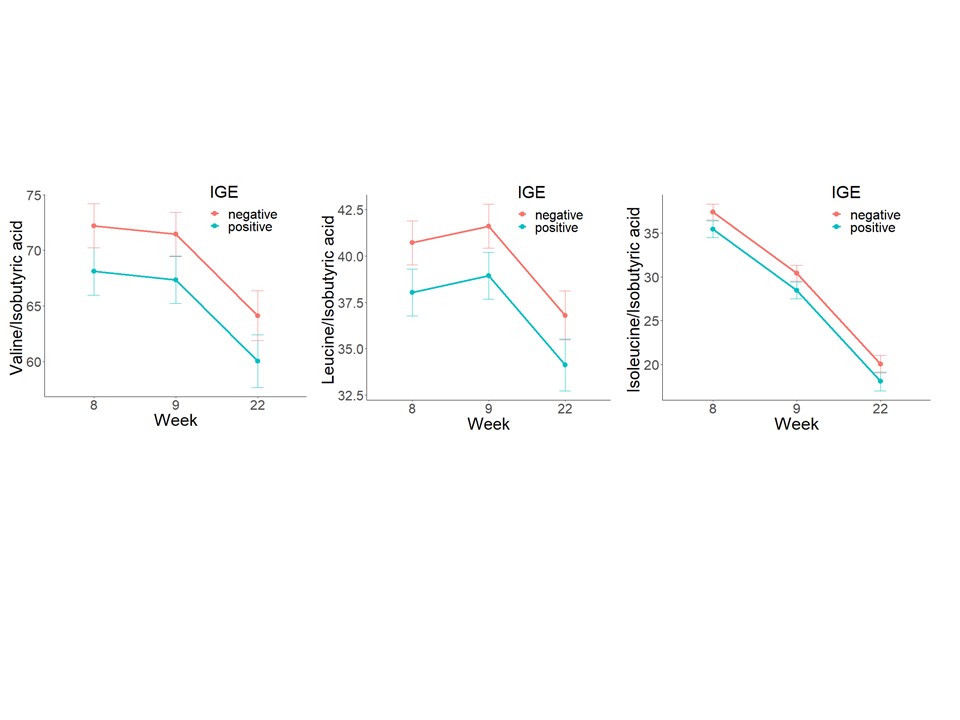

Supplement: Supplementary file 1 — Supplementary Figure S1. [file 41598_2021_2814_MOESM1_ESM.docx]
