# Supplementary material for: Relationship between indirect genetic effects for growth, environmental enrichment, coping style and sex with the serum metabolome profile of pigs
Source: Sci Rep. 2021 Dec 3;11:23377. doi: 10.1038/s41598-021-02814-x (PMC8642533; doi:10.1038/s41598-021-02814-x)

**Supplementary Figure S2**. Partial Least Squared-Discriminant analysis of metabolites concentration of pigs that have an estimated relative positive genetic effect or negative genetic effect (IGE) on the growth of their pen mates. Groups 0, 1 and 2 correspond to samples collected at week 8, 9 and 22 respectively.


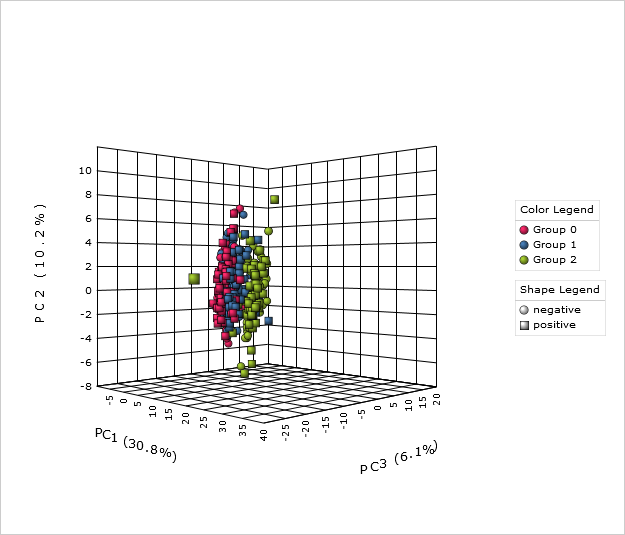

Supplement: Supplementary file 2 — Supplementary Figure S2. [file 41598_2021_2814_MOESM2_ESM.docx]
