# Supplementary material for: Relationship between indirect genetic effects for growth, environmental enrichment, coping style and sex with the serum metabolome profile of pigs
Source: Sci Rep. 2021 Dec 3;11:23377. doi: 10.1038/s41598-021-02814-x (PMC8642533; doi:10.1038/s41598-021-02814-x)

**Supplementary Figure S3.** The effect of interaction (Least squared means and SE) between indirect genetic effect (IGE) on the growth and housing conditions on the magnitude of change (delta) of L-Threonine concentration in serum and on the weight of pigs at week 10.


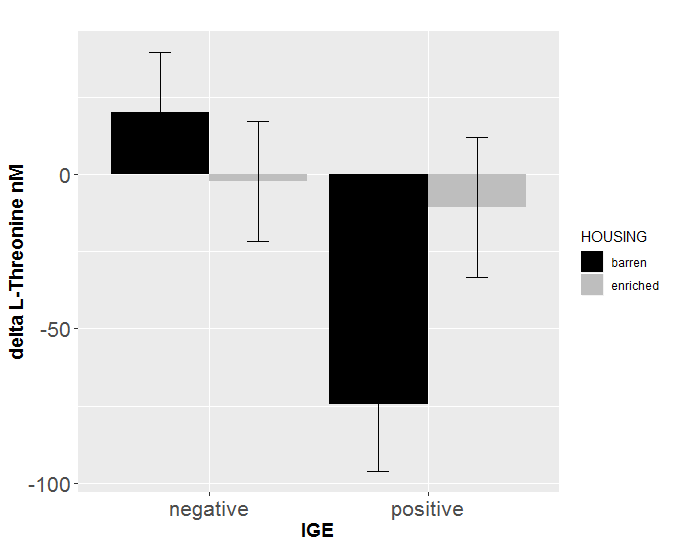

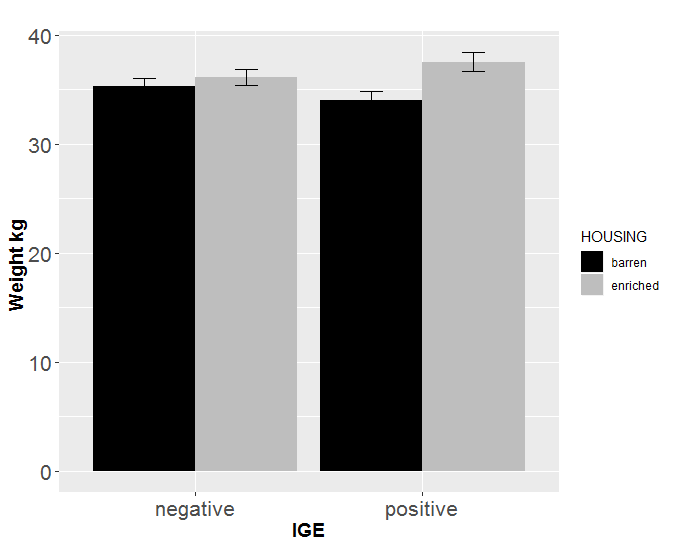

Supplement: Supplementary file 3 — Supplementary Figure S3. [file 41598_2021_2814_MOESM3_ESM.docx]
