# Supplementary material for: Relationship between indirect genetic effects for growth, environmental enrichment, coping style and sex with the serum metabolome profile of pigs
Source: Sci Rep. 2021 Dec 3;11:23377. doi: 10.1038/s41598-021-02814-x (PMC8642533; doi:10.1038/s41598-021-02814-x)
Supplement: Supplementary file 4 — Supplementary Material and Method. [file 41598_2021_2814_MOESM4_ESM.docx]

**Supplementary Material and Method**

**Estimation of indirect genetic effects for growth**

The estimation details of IGE on growth during the finishing phase (from 25 to 110 kg) have been described in Camerlink *et al*.^11^. Briefly, pigs were obtained by mating 64 Topigs-20 sows (Great Yorkshire × Dutch Landrace) and 24 Tempo boars (Great Yorkshire genetic background) with the most extreme positive or negative estimated IGE on growth to create an F1 population (n = 80l). Direct breeding values for growth were kept as similar as possible for both IGE classes. In total, 480 offspring Tempo x Topigs-20 pigs were used. The contrast for estimated IGE between the positive and negative selected offspring was on average 3.6 g/day. With 6 pigs per pen this results in a total contrast of 18 g ADG, ((6−1) × 3.6 = 18 g/day). It was expected that positive IGE offspring would increase the growth of their pen mates, meanwhile negative IGE pigs would decrease the growth of their pen mates. Therefore theoretically, a difference of 2.9 kg in body weight at the end of the finishing phase between positive and negative IGE pigs was expected^11^.

**Animals**

Pigs were born at the experimental farm of Topigs Research Center IPG in Beilen, the Netherlands. Until weaning, they were housed in standard lactation pens of 3.8 m^2^. At 3 days of age males were surgically castrated but tails were not docked. At four weeks of age, the piglets were weaned, and 96 piglets were selected per batch. The pigs were equally divided over five batches. In the present study blood samples of batch 1 and 2 were used for metabolomics analysis. The pigs were housed until slaughter at 23 weeks of age in 16 pens of 6-7 m^2^ located in one room per batch. Each pen consisted of six unrelated pigs, three females and three males. The pigs were also balanced for their IGE scores. All pigs within a pen had either a positive IGE or negative IGE for the growth of their pen mates. In addition, in each pen there were at least two piglets with a high-resisting (HR) or proactive coping style and two with a low-resisting (LR) or reactive coping style^14,20^. Half of the pigs were housed in a barren environment, and the other half in straw enriched environment creating a G × E set-up. The barren pens received two hands of wood shavings every day from 6 weeks of age. The enriched pens contained 1.5 kg of straw and 12 kg of wood shavings. All pens were cleaned daily and afterwards 3 kg of fresh wood shavings and fresh straw (250 g at the start of the experiment and then gradually increased to 1.5 kg) were added to the enriched pens.

**Behavioral tests**

Backtest classification

At two weeks of age, the coping style of the piglets was determined by a backtest. During the backtest, the piglet was put on its back on a table in a room outside its home pen for 60 seconds and manually restrained. The number of struggles, and vocalizations during the test were recorded for each piglet. High-resisters (HR) or the proactive coping style were defined as a piglet that showed two struggles and produced at least 25 vocalizations, or showed at least three struggles. Low-resisters (LR) or the reactive coping style were defined as a piglet that showed zero or one struggle, or two struggles and produced less than 25 vocalizations. Details of the backtest procedure are described in Reimert *et al*.^14^ and Reimert *et al*.^20^.

Regrouping test

Regrouping test details are reported in Camerlink et al.^11^All pigs were subjected to a regrouping test at week 9 of life to induce acute stress and to simulate a similar procedure which takes place in a farm setting. The pigs were regrouped for 24 h with pigs in the same IGE class and housing condition. The new group consisted of three unfamiliar pairs of pigs; each pair was balanced for sex and backtest classification. None of the pigs in the group were full siblings. The new group was relocated to a pen that was unfamiliar to all pigs. After 24 h, the pigs were relocated to their initial pens and reunited with their original pen mates.
