# Supplementary material for: Relationship between indirect genetic effects for growth, environmental enrichment, coping style and sex with the serum metabolome profile of pigs
Source: Sci Rep. 2021 Dec 3;11:23377. doi: 10.1038/s41598-021-02814-x (PMC8642533; doi:10.1038/s41598-021-02814-x)
Supplement: Supplementary file 5 — Supplementary Table S1. [file 41598_2021_2814_MOESM5_ESM.docx]

**Supplementary Table S1**. The number of the animals in each group, batch, indirect genetic effects (IGE), housing, coping style classification and sex

| **Factor** | **Class** | **Batch 1 (n= 92)** | **Batch 2 (n=88)** | **Total** |
| --- | --- | --- | --- | --- |
| IGE | Positive | 48 | 54 | 102 |
|  | Negative | 44 | 34 | 78 |
| Housing | Barren | 47 | 45 | 92 |
|  | Enriched | 45 | 43 | 88 |
| Coping style | HR | 41 | 38 | 79 |
|  | LR | 51 | 50 | 101 |
| Sex | Male | 46 | 44 | 90 |
|  | Female | 46 | 44 | 90 |
