# Supplementary material for: Relationship between indirect genetic effects for growth, environmental enrichment, coping style and sex with the serum metabolome profile of pigs
Source: Sci Rep. 2021 Dec 3;11:23377. doi: 10.1038/s41598-021-02814-x (PMC8642533; doi:10.1038/s41598-021-02814-x)
Supplement: Supplementary file 6 — Supplementary Table S2. [file 41598_2021_2814_MOESM6_ESM.docx]

**Supplementary Table S2**. The number of the variables that were included in the analysis

|  | **Variable name** |
| --- | --- |
| 1 | 1-Methylhistidine |
| 2 | 2-Hydroxybutyrate |
| 3 | 2-Hydroxyisovalerate |
| 4 | 3-Hydroxybutyric acid |
| 5 | 3-Methyl-2-oxovaleric acid |
| 6 | Acetic acid |
| 7 | Acetoacetate |
| 8 | Acetone |
| 9 | Betaine |
| 10 | Citric acid |
| 11 | Choline |
| 12 | Creatine |
| 13 | Creatinine |
| 14 | D-Glucose |
| 15 | Dimethylglycine |
| 16 | Dimethyl sulfone |
| 17 | Ethanol |
| 18 | Formate |
| 19 | Glycerol |
| 20 | Glycine |
| 21 | Hypoxanthine |
| 22 | Isobutyric acid |
| 23 | Isoleucine |
| 24 | Isopropanol |
| 25 | Ketoleucine |
| 26 | L-Alanine |
| 27 | L-Alpha-aminobutyric acid |
| 28 | L-Arginine |
| 29 | L-Asparagine |
| 30 | L-Aspartic Acid |
| 31 | L-Carnitine |
| 32 | L-Glutamic acid |
| 33 | L-Glutamine |
| 34 | L-Histidine |
| 35 | L-Lactic acid |
| 36 | L-Leucine |
| 37 | L-Lysine |
| 38 | L-Methionine |
| 39 | L-Ornithine |
| 40 | L-Phenylalanine |
| 41 | L-Proline |
| 42 | L-Serine |
| 43 | L-Threonine |
| 44 | L-Tyrosine |
| 45 | L-Valine |
| 46 | Mannose |
| 47 | Methanol |
| 48 | Pyruvic acid |
| 49 | Succinate |
| 50 | Glucogenic AA |
| 51 | Ketogenic AA |
| 52 | BAA |
| 53 | Ketone bodies |
| 54 | Creatine/Creatinine |
| 55 | L-Aspartic acid/L-Glutamic acid |
| 56 | L-Isoleucine/Isobutyric acid |
| 57 | L-Leucine/Isobutyric acid |
| 58 | L-Valine/Isobutyric acid |
| 59 | Epinephrine |
| 60 | Phenylethylamine |
| 61 | Tyramine |
| 62 | Serotonin |
| 63 | Vitamin B2 (Riboflavin) |
| 64 | Vitamin B5 (Pantothenic acid) |
| 65 | Vitamin B7 (Biotin) |

Variables 1-49 are metabolites that were quantified using Nuclear Magnetic Resonance (NMR). Variables 50-58 are indexes and ratios that were calculated. Glucogenic AA were calculated as the sum of all the amino acids, except L-leucine and L-lysine. Ketogenic AA were calculated as the sum of L-leucine and L-lysine. Branched AA (BAA) index was calculated as the sum of L-valine, L-leucine, and L-isoleucine. Ketone bodies was calculated as the sum of 3-Hydroxybutyric acid, acetoacetate and acetone. Variables 59-62 are catecholamines and neurotransmitter. Variables 63-65 are water soluble vitamins.
